# Adipose tissue quantity, distribution and pathology and its relationship with type-2 diabetes, insulin resistance and other clustering disease risk in South Asians: a cross-sectional study

**DOI:** 10.3389/fendo.2025.1622732

**Published:** 2025-09-15

**Authors:** Aditya Saxena, Sanjay Saran, Praveen Choudhary, Balram Sharma, Shalu Gupta, Rajendra Mandia, Ramesh C. Banshiwal, Ravinder Kumar Lamoria, Anurag Dhakad, Utkarsh Raj, Pradeep Tiwari, Sandeep Kumar Mathur

**Affiliations:** ^1^ Department of Bioinformatics, Faculty of Sciences, Marwadi University, Rajkot, India; ^2^ Department of Endocrinology, Sawai Man Singh (SMS) Medical College, Jaipur, India; ^3^ Department of General Surgery, Sawai Man Singh (SMS) Medical College and Attached Hospital, Jaipur, India; ^4^ Department of Orthopedics, Sawai Man Singh (SMS) Medical College and Attached Hospital, Jaipur, India; ^5^ Department of Biotechnology & Bioinformatics, NIIT University, Neemrana, Rajasthan, India; ^6^ Department of Biotechnology and Bioinformatics, Birla Institute of Scientific Research, Jaipur, India

**Keywords:** type 2 diabetes, insulin resistance, metabolic syndrome, visceral adiposity, ectopic liver fat, adipocyte hypertrophy, South Asians

## Abstract

**Background:**

South Asians exhibit distinct metabolic characteristics that may predispose them to insulin resistance (IR), type 2 diabetes (T2D), and metabolic syndrome (MetS). Adipose tissue dysfunction, including abnormal fat distribution and inflammatory processes, has been implicated in metabolic deterioration. However, the specific contributions of adipocyte hypertrophy, ectopic fat accumulation, and immune-related changes remain unclear. Understanding these mechanisms is essential for refining metabolic health interventions.

**Method:**

A total of 322 individuals (110 diabetics, 212 non-diabetics) underwent comprehensive clinical, biochemical, and radiological assessments. Body fat distribution, visceral fat, and ectopic liver fat were quantified using dual-energy X-ray absorptiometry (DEXA) and magnetic resonance imaging (MRI). Adipose biopsies were performed to examine adipocyte size and macrophage infiltration across subcutaneous (SAT), visceral (VAT), and femoral fat depots. Metabolic parameters, including MetS score and homeostatic model assessment for insulin resistance (HOMA-IR), were correlated with adipose characteristics.

**Results:**

compared to non-diabetics, diabetics demonstrated significantly higher body mass index (BMI), waist-to-hip ratio, total fat mass, and ectopic liver fat (*P* < 0.05). While visceral and lower limb fat masses were similar after BMI adjustment, ectopic liver fat remained markedly elevated in diabetics (*P* = 0.002). Adipocyte hypertrophy was detected in visceral and femoral fat (*P* = 0.01), with an increased inflammatory macrophage-ratio (M1/M2) observed in subcutaneous fat (*P* = 0.006). Strong correlations were identified between MetS score, HOMA-IR, BMI, visceral adipocyte size, ectopic liver fat, and macrophage ratio (*P* < 0.001). However, regional adipose mass lost its correlation with IR and MetS after adjusting for adipocyte size. Inflammatory markers and adipose dysfunction appeared to be central to metabolic deterioration.

**Conclusion:**

These insights suggest that interventions aimed at reducing adipocyte hypertrophy, ectopic fat accumulation, and adipose inflammation may offer more targeted strategies for improving metabolic health in South Asians.

## Introduction

Type-2 diabetes (T2D) and metabolic syndrome (MetS) in South Asians show a unique clinical phenotype which is different as compared to that of Caucasians and other races (1–4). Their distinguishing features include relatively lower body mass index (BMI), higher percentage body fat, predominately central fat deposition (visceral adiposity), high insulin resistance (IR), heightened T2D, atherosclerotic cardiovascular disease (ASCVD) and other clustering disease risks. Traditionally, a higher degree of dysmetabolism in them is attributed to the relatively higher visceral fat content (5). However, the adiposity and dysmetabolism relationship is complex and multi-faceted and it remains largely unexplored, particularly in this population (6).

This paradox—relatively lower levels of total adiposity yet disproportionately higher metabolic risk—raises critical questions about the underlying mechanisms contributing to cardiometabolic disease in South Asians.

The three important characteristics of adipose tissue that could potentially contribute to IR, T2D, and other clustering disease risks are (1) total body fat quantity (measured as BMI or whole-body percentage body fat content), (2) fat quantity in regional depots like abdominal subcutaneous (SAT), intraperitoneal adipose depots surrounding the vital organs (IPAT-SV), peripheral depots like gluteo-femoral (FAT) and ectopic fat deposition in liver, muscle, pancreas etc. (7), (3) pathological qualitative alterations in adipose tissue characterized by adipocyte hypertrophy, inflammatory macrophage infiltration with altered polarity from M2 to M1 (assessed as adipocyte size and number and M1/M2 macrophage ratio), fibrosis etc (8).

Adipocyte hypertrophy refers to an increase in the size of existing fat cells as they store more lipids. Adipocyte hyperplasia, on the other hand, is the process of increasing the number of adipocytes through the proliferation and differentiation of precursor cells. These are two distinct mechanisms of adipose tissue expansion.

Several recent studies have investigated the relationship between different facets of adipose tissue in isolation (*i.e.*, its quantity, distribution, and pathological changes) with IR, T2D and other clustering disease risks in South Asians. Diabetics as compared to non-diabetics have been found to contain higher and lower quantities of fat respectively in their visceral and peripheral compartments (9, 10). Therefore, the higher IR and dysmetabolism risk in them has traditionally been attributed to this fat distribution pattern (11). However, we recently found that insulin resistance in these individuals is more closely linked to the amount of ectopic liver fat than with visceral adipose tissue mass (*i.e.*, the intraperitoneal adipose tissue surrounding the vital organs) (12). Moreover, in the higher quartiles of IR, this relationship became further stronger. Additionally, in its highest quartile, IR shows a negative correlation with the abdominal subcutaneous adipose tissue mass.

These observations challenge the prevailing view that visceral fat is the primary driver of metabolic dysfunction in South Asians. Instead, they shift attention toward ectopic lipid accumulation and subcutaneous fat insufficiency as key contributors to insulin resistance.

Several studies have investigated the relationship between the qualitative traits of adipose tissue like adipocyte size with T2D, IR, and other clustering diseases in South Asians (13, 14). South Asians were found to have larger adipocyte areas as compared to white Caucasians (14). This difference in adipocyte area has also been reported to account for the ethnic differences in insulin, HDL cholesterol, adiponectin, and ectopic fat deposition in the liver as compared to white Caucasians (15). Furthermore, in these individuals, weight gain was linked to a greater accumulation of ectopic liver fat and a more pronounced decline in insulin sensitivity compared to Caucasians. Weight gain was also not associated with a major change in the number of small adipocytes (16).

These findings are consistent with the “nutrient overflow hypothesis,” wherein a limited capacity of protective fat depots (such as subcutaneous tissue) leads to triglyceride spillover into non-adipose organs like the liver and muscle, contributing to metabolic deterioration. In other words, all the above findings support the nutrient overflow hypothesis of their “thin fat phenotype” (17, 18).

This hypothesis is further supported by the findings of the studies investigating the relationship between the transcriptome in various adipose tissue depots with T2D and its intermediate phenotypic traits (19–21). An interesting finding of these studies is that the differentially expressed genes in various adipose depots in diabetics not only enrich the pathways of adipogenesis, inflammation, and several other known aspects of the pathological adipose tissue, but their modules of co-expressed genes showed an association with several intermediate phenotypic traits of T2D. Therefore, these findings support the concept of “adiposopathy” as a disease playing a major role in the pathogenesis of IR and T2D in South Asians (22).

Despite this growing body of evidence, a cohesive picture outlining how these diverse adipose tissue characteristics—quantity, distribution, and quality—interact to influence metabolic risk remains elusive. This is particularly important in South Asians, where conventional obesity metrics may fail to capture the true burden of metabolic dysfunction.

Therefore, in the present study, we adopted an integrative approach to examine the relationship between quantitative (total body fat), regional (including ectopic), and pathological (morphological and inflammatory) traits of adipose tissue with IR, T2D, and clustering metabolic diseases (MetS) in a South Asian cohort.

## Methodology

### Study subjects

This is a cross-sectional analysis of the data of the individuals who participated in a study on the clinical, biochemical, and cellular correlates of the transcriptome of adipose tissue biopsies obtained from peripheral and central adipose tissue depots in Asian Indian T2D individuals and its subsequent qPCR and clinical validation studies sharing common inclusion and exclusion criteria and clinical biochemical, radiological and adipose tissue cellular phenotyping protocol. These studies were conducted at a tertiary care hospital in northwest India. The study enrolled two groups of participants: (1) individuals with T2D, diagnosed based on the American Diabetes Association (ADA) criteria, and (2) non-diabetic controls undergoing elective surgery for non-infectious, non-malignant conditions. Within each group, participants were further categorized into two subgroups based on the type of surgery: (a) those undergoing abdominal procedures (e.g., cholecystectomy, hernia repair), and (b) those undergoing thigh surgeries (e.g., fracture fixation, trauma management). Individuals currently receiving medications known to affect insulin resistance or adipocytokine expression—including thiazolidinediones, metformin, and glucocorticoids—were excluded from the study.

A total of 322 individuals (110 diabetics and 212 non-diabetics) with a 1:1 male-to-female ratio were included in this study. The majority of them were non-obese (BMI < 30). A total of 143 individuals (56 T2DM, 87 non-diabetics) were undergoing abdominal surgery, and 179 (54 T2DM, 125 non-diabetics) were undergoing thigh surgery.

### Biochemical and clinical assessment

Anthropometric assessments were conducted for all subjects, measuring body weight, height, waist circumference, and the waist-to-hip (W:H) ratio. BMI of the subjects was calculated by using the formula BMI = (weight in kg)/(height in meters)^2^. Cutaneous markers of insulin resistance: acrochordons and acanthosis nigricans were assessed in all participants. Supine blood pressure was assessed using a mercury sphygmomanometer (BPMR-120 Diamond Delux, Industrial Electronic and Allied Products, Maharashtra, India) following a 5-minute rest period. Blood samples were collected at 8:00 a.m. following a minimum of 8 hours of overnight fasting. Biochemical assessments included measurements of serum glucose, triglycerides, low-density lipoprotein cholesterol (LDL), high-density lipoprotein cholesterol (HDL), and very-low-density lipoprotein cholesterol (VLDL) using the Kopran AU/400 fully automated analyzer (Olympus Corporation, Shinjuku, Tokyo, Japan). Serum insulin was analyzed via a chemiluminescent immunometric assay using the Immulite 2000 (Siemens Healthineers AG, Erlangen, Germany). HbA1c levels were determined through the turbidimetry method with BioSystems (Biosystems, S.A. Barcelona, Spain) kits. HOMA-β was computed using the following formula:


$$\frac{360\;x\;\left[Insulin\;in\;µU/ml\right]}{\left[Glucose\;in\;mg/dL\right]-63}$$

Similarly, HOMA-IR, an indicator of insulin resistance, was determined using the formula:


$$\frac{\left[Glucose\;in\;mg/dL\right]\;x\;\left[Insulin\;in\;µU/ml\right]}{405}$$

The term MetS score is used in this paper for the number of components of MetS present in an individual and the components of MetS were defined as those recommended by ATP III.

### Radiological assessment

Distribution & content of body fat was recorded for 102 subjects undergoing femoral surgery and 75 subjects undergoing abdominal surgery by ‘Dual-Energy X-ray Absorptiometry (DEXA)’ using the ‘S/N91395 Hologic Explorer model (Hologic Canada ULC, Mississauga, Ontario, Canada)’.

Magnetic resonance imaging (MRI) was done on 101 subjects undergoing abdominal surgery and 67 subjects undergoing femur bone surgery. Ectopic liver, abdominal visceral (VAT) and subcutaneous adipose tissue (SAT) masses were calculated.

MRI scan procedure: MRI scans were performed using the Philips Ingenia 3-Tesla machine. The observer and the radiologist (Single Radiologist) who interpreted the scans on Osirix software were unaware of the clinical status of the study subjects. A single scan (3 mm) of the abdomen was done at the level of L4-L5 vertebrae and analyzed for a cross-sectional area of adipose tissue, which was expressed in centimeters squared. The parameters studied included VAT and SAT. VAT, which represents intra-abdominal omental fat (without ectopic fat) was distinguished from SAT by tracing along the fascial plane defining the internal abdominal wall and the area was calculated in centimeters square. Ectopic liver fat was measured using liver intensity on Osirix software using Dixon method (Liver Fat percentage = 100x (Signal intensity liver/signal intensity spleen) on in-phase T1- (signal intensity liver/signal intensity spleen) on outphase T1)/2X(signal intensity liver/signal intensity spleen) on inphase T1) (23).

MRI was performed on 144 visceral tissue samples, including 93 from diabetic subjects and 51 from non-diabetic subjects, 138 subcutaneous tissue samples (90 diabetics, 48 non-diabetics), and 137 femoral tissue samples (87 diabetics, 47 non-diabetics).

### Adipose tissue biopsy

Adipose tissue biopsies were obtained from participants undergoing femoral bone surgery as well as undergoing abdominal surgery (two biopsy samples from each subject, one from SAT and one from VAT; total 143-pairs). Each adipose tissue biopsy was divided in two samples. One sample was stored in Qiagen All Protect tissue reagent (Cat. No. 76405) and the other was stored in formalin solution. Samples preserved in Qiagen All Protect tissue reagent were processed for genome-wide gene expression profiling studies, whereas those stored in formalin solution were prepared as tissue blocks and slides for adipocyte size analysis. Following parameters were estimated on these adipose tissue samples:

#### Adipose tissue cell size measurement

It was conducted using biopsies preserved in formalin solution, which were processed into tissue blocks and slides following standard protocols. Adipocyte imaging was performed at 10× magnification with the Motic Panthera Moticam 5 trinocular microscope (BA210LED) (Motic Incorporation Ltd., Hong Kong, China). The adipocyte area was quantified in square micrometers (µm²) using the ImageJ image analysis tool (http://imagej.nih.gov/ij). cell size measurements were obtained from 148 visceral tissue samples (97 diabetics, 41 non-diabetics), 112 subcutaneous tissue samples (79 diabetics, 33 non-diabetics), and 51 femoral tissue samples (37 diabetics, 14 non-diabetics).

#### Adipose tissue cell phenotype/immune-histochemistry

The number and the type of infiltrating macrophages (i.e. M1 and M2) were estimated by immunohistology-chemistry. They were counted in ten randomly selected areas at 40x magnification. The number of macrophages was normalized to 100 adipocytes. CD80 (Thermo Fisher Scientific) and CD163 (Thermo Fisher scientific) markers were used for macrophage labelling. M1/M2 macrophage ratio assessments were conducted on 22 visceral tissue samples (22 diabetics, 9 non-diabetics) and 40 subcutaneous tissue samples (31 diabetics, 9 non-diabetics).

### Statistical analysis

Comparison of variables, which followed normal distribution was done by ‘Student’s t-test’. Variables that did not have a normal distribution were compared using the Mann-Whitney Wilcoxon Test. Correlations of femoral, omental, and subcutaneous adipocyte size with body composition and metabolic parameters were evaluated by Spearman’s Rank-sum correlation coefficient. Statistical analysis was carried out using the Python library - SciPy which is extensively used for statistics, optimization, linear algebra, and integration, and a *P*-value of 0.05 was taken as statistically significant.

## Results

### General characteristics

The study included 322 individuals, comprising 212 non-diabetics and 110 diabetics (**Table 1**). Diabetics were marginally older (51.42 ± 15.06 vs. 56.24 ± 10.89, t = -2.974 *P* = 0.03). Most participants were non-obese, with an average BMI of 23.6, and the male-to-female ratio was 1:1. Of the total, 225 individuals scored 0–2 for MetS score (*i.e.,* number of components of MetS as per ATP-III criteria), while 149 scored 3-5. Visceral adipocyte showed a moderate to strong positive correlation with subcutaneous adipocyte (*r* = 0.585, *P* < 0.001), while visceral M1/M2 and subcutaneous M1/M2 macrophage polarization indices were moderately correlated (*r* = 0.503, *P* < 0.001), suggesting coordinated metabolic and immune remodeling across adipose depots.

**Table 1:** t-Test analysis between diabetics and nondiabetics across measured anthropometric, cellular, and biochemical traits.

S. No.	Traits	Non-Diabetics (Mean ± SD)	Diabetics (Mean ± SD)	t-statistic	P-value
1	BMI	23.03 ± 4.12	24.77 ± 5.20	-3.262	**0.001**
2	Waist circumference (c.m.)	85.00 ± 10.20	93.84 ± 9.94	-7.412	**>> 0.001**
3	W:H	0.92 ± 0.10	0.98 ± 0.10	-5.064	**>> 0.001**
4	Total Cholesterol (mg/dL)	166.37 ± 43.97	186.48 ± 49.12	-3.7	**0.0002**
5	Triglycerides (mg/dL)	137.82 ± 72.07	160.03 ± 62.39	-2.723	**0.007**
6	HDL (mg/dL)	45.48 ± 12.34	43.46 ± 9.58	1.487	0.138
7	LDL (mg/dL)	84.65 ± 29.38	101.40 ± 35.19	-4.468	**>> 0.001**
8	VLDL (mg/dL)	27.71 ± 16.50	34.88 ± 21.70	-3.253	**0.001**
9	HOMA-B	131.51 ± 243.26	79.11 ± 88.54	2.184	**0.029**
10	HOMA-R	1.76 ± 1.83	8.79 ± 8.68	-11.311	**>> 0.001**
11	Insulin (mU/dL)	7.61 ± 6.82	18.49 ± 14.28	-9.228	**>> 0.001**
12	Glucose fasting (mg/dL)	91.39 ± 15.93	185.84 ± 77.77	-16.993	**>> 0.001**
13	Hb1Ac (%)	5.28 ± 0.73	8.37 ± 1.78	-21.784	**>> 0.001**
14	MetS Score	1.80 ± 1.17	3.66 ± 1.04	-14.062	**>> 0.001**

Bold font indicates statistically significant values.

### Comparison of clinical & biochemical parameters between diabetics and non-diabetics

**Table 1** presents the comparative baseline anthropometric and biochemical parameters between diabetic and non-diabetic individuals in the study cohort. As expected, diabetics had significantly higher BMI (*P* = 0.001), waist circumference (*P* < 0.001), and waist-to-hip ratio (*P* < 0.001), indicating a greater degree of central adiposity.

In terms of lipid profiles, diabetics exhibited elevated levels of total cholesterol (*P* = 0.0002), triglycerides (*P* = 0.007), LDL (*P* < 0.001), and VLDL (*P* = 0.001) compared to their non-diabetic counterparts.

Glycemic markers and insulin resistance indices were markedly worse in diabetics, who displayed significantly higher fasting glucose, insulin, HOMA-R, and HbA1c levels (all *P* < 0.001). These findings corroborated the expected dysmetabolic profile associated with T2D and establish the foundation for evaluating adipose tissue correlates in subsequent analyses.

### Comparison of parameters of adipose tissue content, distribution and qualitative traits between diabetics and non-diabetics

**Table 2** presents the distribution of adipose tissue across key regional depots. Compared to non-diabetics, diabetics were found to have higher total body fat (*P* = 0.001), trunk fat (*P* = 0.002), and combined lower limb fat mass (*P* = 0.008). Additionally, they exhibited increased visceral fat (*P* = 0.02) and ectopic liver fat mass (*P* = 6.17 × 10^-5^). Upon correction for BMI, differences in visceral and lower limb fat mass became non-significant, but ectopic liver fat mass remained significantly higher (*P* = 0.002). Trends towards higher visceral adipocyte size (*P* = 0.05), femoral adipocyte size (*P* = 0.01), and subcutaneous adipocyte size (*P* = 0.33) were observed among diabetics. The M1:M2 ratio showed a trend towards being higher in visceral and femoral adipose depots, and significantly higher in the subcutaneous compartment. Despite exhibiting the highest M1/M2 macrophage ratio within femoral adipose tissue as compared to visceral, and subcutaneous fat, the difference between diabetic and non-diabetic individuals was not statistically significant (*P* < 0.79). This finding suggests that, in Asian Indians, peripheral adipose depots may exhibit a pro-inflammatory milieu even in the absence of overt diabetes, thereby reinforcing the concept of “sick fat” as a preclinical hallmark of metabolic dysfunction. The visceral adipocyte and fat size ratio were significantly lower in diabetics, indicating possible adipocyte hypertrophy or impaired recruitment (*P* = 0.024). This analysis suggested that BMI and adipocyte hypertrophy contribute to observed differences in fat distribution between diabetics and non-diabetics. (**Table 2**).

**Table 2:** t-Test analysis between diabetics and nondiabetics across measured DEXA, and MRI traits for fat quantity, distribution.

S. No.	Traits	Non-Diabetics (Mean ± SD)	Diabetics (Mean ± SD)	t-statistic	P-value
1	Subcutaneous adipocyte size	12302.79 ± 6853.72	1355.46 ± 7719.94	-0.9	0.33
2	Visceral adipocyte size	12757.57 ± 6485.53	14911.66 ± 6558.03	-1.9	0.05
3	Femoral adipocyte size	8037.31 ± 4996.61	10270.67 ± 5901.98	-2.4	**0.01**
4	M1:M2 ratio (Subcutaneous)	0.29 ± 0.22	0.87 ± 1.23	-2.89	**0.006**
5	M1:M2 ratio (Visceral)	0.46 ± 0.33	0.66 ± 0.57	-1.69	0.096
6	M1:M2 ratio (Femoral)	0.88 ± 0.65	1.64 ± 1.13	-1.83	0.079
7	Total Fat Mass (g)	17395.64 ± 8193.10	22172.16 ± 10140.03	-3.3	**0.001**
8	Total Fat Mass (g) corrected for BMI	855.80 ± 400.12	913.68 ± 430.23	-1.30	0.195
9	Trunk Fat Mass (g)	8368.35 ± 5588.35	11090.3 ± 6158.15	-3.02	**0.002**
10	Trunk Fat Mass (g) corrected for BMI	450.12 ± 200.34	480.56 ± 220.45	-1.22	0.224
11	Upper Limb Fat Mass (g)	2313.26 ± 1419.74	2761.34 ± 1915.21	-1.7	0.085
12	Upper limb Fat Mass (g) corrected for BMI	85.23 ± 45.67	92.45 ± 50.12	-1.34	0.182
13	Lower Limb Fat Mass (g) (L+R)	5669.65 ± 2455.86	6934.9 ± 3486.17	-2.69	0.008
14	Lower limb Fat Mass (g) corrected for BMI	320.45 ± 150.23	340.67 ± 160.34	-1.15	0.251
15	Subcutaneous fat mass (cm^2^)	134.61 ± 53.68	158.48 ± 95.2	-1.8	0.06
16	Subcutaneous fat mass (cm^2^) corrected for BMI	5.67 ± 2.98	6.12 ± 3.21	-1.23	0.221
17	Visceral Fat mass (cm^2^)	88.73 ± 40.56	154.6 ± 67.5	2.28	**0.024**
18	Visceral fat mass (cm^2^) corrected for BMI	6.12 ± 3.45	7.89 ± 4.12	-2.45	**0.016**
19	Ectopic liver fat signal intensity	6.37 ± 3.01	9.4 ± 4.7	-4.2	**6.17 X 10^-5^**
20	Ectopic liver fat signal intensity corrected for BMI	0.23 ± 0.12	0.35 ± 0.18	-3.12	**0.002**
21	Visceral Fat mass corrected for adipocyte size	0.01 ± 0.02	0.01 ± 0.01	-0.42	0.67
22	Subcutaneous Fat mass corrected for adipocyte size	0.01 ± 0.01	0.01 ± 0.01	-0.02	0.98
23	Lower limb Fat mass corrected for adipocyte size	0.83 ± 0.53	0.89 ± 0.48	-0.63	0.53

Bolded values represent statistically significant results (*P* < 0.05).

### Correlation analysis for HOMA-IR and MetS score across measured anthropometric, cellular, and biochemical traits

**Table 3** presents the correlation coefficients and significance values between metabolic syndrome MetS scores, HOMA-IR, and various adiposity traits. Higher MetS scores were strongly associated with non-corrected parameters such as BMI (*P* = 9.37 × 10^-7^), waist circumference (*P* < 0.001), and waist-to-hip ratio (*P* = 1.18 × 10^-8^). However, BMI-corrected traits demonstrated weaker or non-significant correlations. For example, total body fat mass corrected for BMI (*P* = 0.381) and trunk fat mass corrected for BMI (*P* = 0.617) showed negligible associations, indicating the dominant influence of BMI itself on these traits.

**Table 3:** Correlation analysis for HOMA-IR and MetS score across measured anthropometric, cellular, and biochemical traits.

S. No.	Trait	HOMA-R	HOMA-R *P*-value	MetS	MetS *P-*value
1	BMI	0.16	3.05 × 10^-3^	0.26	**9.37 × 10^-7^**
2	Waist circumference	0.28	1.37 × 10^-7^	0.56	**≈ 0**
3	Waist-to-Hip	0.17	1.35 × 10^-3^	0.31	**1.18 × 10^-8^**
4	Total body fat mass	0.13	0.065	0.21	**0.003**
5	Total body fat mass corrected for BMI	0.064	0.39	0.066	0.381
6	Trunk fat mass	0.127	0.091	0.16	**0.032**
7	Trunk body fat mass corrected for BMI	0.07	0.35	0.038	0.617
8	Upper limb fat mass	0.059	0.43	0.18	0.43
9	Upper limb fat mass corrected for BMI	0.0002	0.998	0.063	0.398
10	Lower limb fat mass	0.12	0.091	0.24	**0.001**
11	Lower limb fat mass corrected for BMI	0.027	0.72	0.081	0.277
12	Visceral Adipocyte Size	0.2	1.75 × 10^-4^	0.27	**3.96 × 10^-7^**
13	Peripheral Adipocyte Size	0.21	1.08 × 10^-4^	0.23	**2.14 × 10^-5^**
14	Subcutaneous adipocyte size	0.4	0.004	0.41	**0.004**
15	M1/M2 macrophage ratio in Visceral Fat	-0.11	0.42	-0.17	0.23
16	M1/M2 macrophage ratio in Subcutaneous Fat	-0.1	0.48	-0.13	0.37
17	M1/M2 macrophage ratio in Peripheral Fat	0.23	**2.19 × 10^-5^**	0.32	**3.79 × 10^-9^**
18	Visceral Fat mass	0.13	**1.71 × 10^-2^**	0.23	**2.14 × 10^-5^**
19	Visceral Fat mass corrected for BMI	0.041	0.604	-0.021	0.79
20	Subcutaneous Fat Mass	0.2	0.13	0.47	**0.0008**
21	Subcutaneous Fat mass corrected for BMI	-0.047	0.55	0.116	0.143
22	Ectopic Fat mass	0.33	1.26 × 10^-9^	0.27	**6.07 × 10^-7^**
23	Ectopic Fat Mass corrected for BMI	0.217	0.006	0.213	**0.007**
24	Visceral fat mass corrected for adipocyte size	-0.09	0.52	-0.08	0.58
25	Subcutaneous fat mass corrected for adipocyte size	-0.1	0.48	-0.05	0.7
26	Peripheral fat mass corrected for adipocyte size	0.24	0.03	0.56	**< 0.001**

Bolded values represent statistically significant results (*P* < 0.05).

Similarly, HOMA-IR showed significant correlations with non-corrected traits like BMI (*P* = 3.05 × 10^-^³), waist circumference *(P* = 1.37 × 10^-7^), and waist-to-hip ratio (*P* = 1.35 × 10^-^³), while BMI-corrected parameters, such as total body fat mass corrected for BMI (P = 0.39) and visceral fat mass corrected for BMI *(P* = 0.604), displayed weaker or non-significant relationships.

Interestingly, visceral and subcutaneous fat masses corrected for adipocyte size showed minimal correlation with MetS (*P* = 0.58 and *P* = 0.70, respectively) and HOMA-IR, suggesting that total fat mass alone is less informative than cellular-level pathology. This unconventional inverse correction aimed to discern whether metabolic risk is more closely linked to depot volume or to adipocyte hypertrophy. In contrast, visceral adipocyte size was strongly associated with both MetS (*P* = 3.96 × 10^-7^) and HOMA-IR (*P* = 1.75 × 10^-4^), underscoring the central role of adipocyte enlargement in metabolic dysfunction. These findings further suggest that uncorrected adiposity traits—such as BMI and fat distribution—exhibit stronger associations with metabolic syndrome and insulin resistance than BMI-corrected indices, highlighting the independent contribution of overall obesity and depot-specific characteristics to metabolic risk.

## Discussion

This study investigated the relationship between certain parameters of generalized adiposity, regional fat depot masses, and adipose tissue pathology in some of these depots with T2D, IR, and MetS in a cross-sectional design. Diabetics were found to have higher BMI, W:H ratio, waist circumference, total body, trunk, lower limb, visceral, and ectopic liver fat masses. However, when corrected for BMI, the most of regional fat depot masses were found to be comparable with controls, except the ectopic liver fat mass. Diabetes also had larger adipocyte size and borderline high M1/M2 ratio in the visceral and the lower limb adipose depots. When corrected for the adipocyte size, the higher adipose tissue masses in these two compartments were also attenuated and became comparable with those of controls. Several of these parameters, like those for generalized obesity (*i.e.*, BMI), regional adipose deposition (W:H ratio, waist circumference, visceral, subcutaneous, lower limb, and ectopic liver fat mass), and quality of adipose tissue (adipocyte size in all the studied compartments, M1:M2 ratio in lower limbs) showed a significant association with IR and MetS. However, when corrected for adipocyte size, the quantitative traits of adipose tissue mass in the visceral and subcutaneous compartment did not show any significant correlation with IR and MetS.

**Figure 1:**
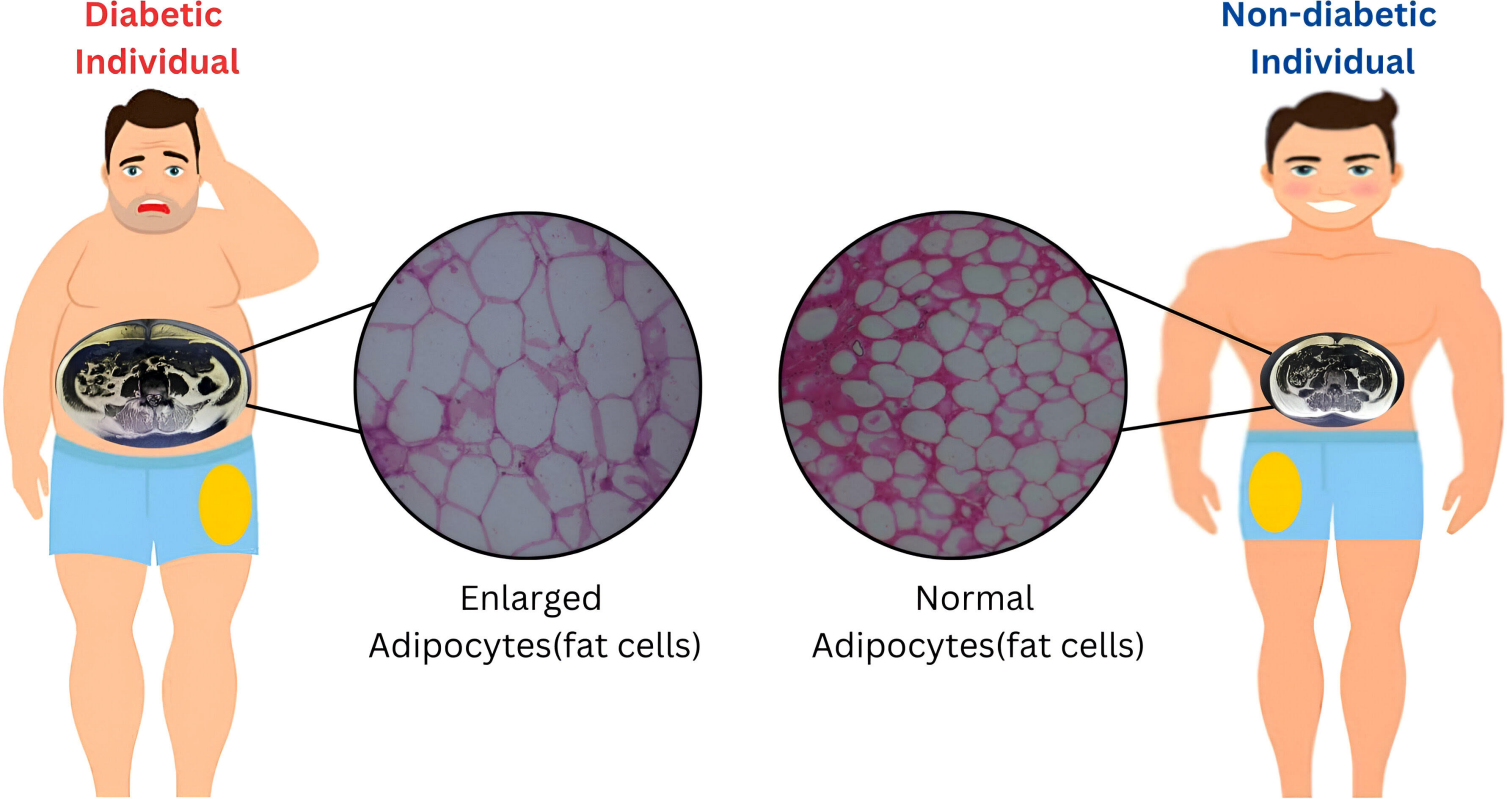
Adipocyte Hypertrophy Drives Regional Fat Distribution and Its Role in T2D, IR, and MetS.

Although a statistically significant increase in adipocyte size was observed primarily in the femoral depot of individuals with T2D, a similar trend toward hypertrophy was also evident in the visceral fat compartment (*P* = 0.05). While subcutaneous adipocyte size did not differ significantly between groups, the overall pattern—particularly within metabolically active fat depots—suggests that the greater fat mass observed in diabetics may result predominantly from adipocyte hypertrophy rather than hyperplasia. Taken together, these findings (**Figure 1**) indicate that total and regional fat accumulation in individuals with type 2 diabetes is more likely to reflect an expansion in adipocyte size. This supports the hypothesis that insulin resistance (IR) and metabolic syndrome (MetS) in these individuals may, in part, be driven by adipocyte hypertrophy and ectopic fat deposition in the liver.

Therefore, among the quantitative (both whole body and regional) and qualitative adipose traits studied here, adipocyte size and ectopic liver fat are possibly the most important adipose trait showed an association with T2D, IR and MetS.

High IR and T2DM risk among Asian Indians have traditionally been attributed to the higher fat quantity in their visceral adipose tissue compartment (2, 24, 25). On the contrary peripheral and abdominal subcutaneous adipose tissue depots are considered protective (26, 27). The present study, which additionally probed the qualitative aspects of these adipose depots, suggests that the quantitative traits of excess visceral adipose tissue mass reflect adipocyte hypertrophy, a qualitative trait of adipose tissue pathology, which also shows an association with IR and MetS. Contrary to conventional belief, diabetics also had a higher quantity of fat in their lower limbs, and the adipocyte hypertrophy in this depot also showed a positive association with IR and MetS (28–32). Therefore, these findings are not consistent with the belief that accumulation of fat in the lower body (gluteo-femoral obesity) shows opposite associations with cardiovascular disease and type 2 diabetes mellitus when adjusted for overall fat mass (33, 34). One explanation for this contradictory finding of the present study is that in diabetics, it is the failure of gluteo-femoral fat to perform its physiological function of fat accumulation that results in a qualitative change of adipocyte hypertrophy, inflammation, and ectopic fat deposition in the liver. These qualitative adipose traits possibly play an important role in the pathogenesis of IR, T2D, and MetS. This explanation is supported by our previous reports on transcriptomic profiling of femoral adipose tissue in diabetics where the differentially expressed genes not only showed an association with T2D and its intermediate phenotypic traits, but also converges on the known aspects of the pathologic adipose tissue (*i.e.*, sick fat) like adipogenesis defects and inflammation (20, 35).

The findings of this study are to some extent consistent with the nutrient overflow hypothesis of “Asian Indian thin fats” (2). According to this theory, in the states of positive calorie balance, when the capacity of subcutaneous compartments is exhausted, the excess fat gets deposited in the visceral and ectopic fat compartments. What the present study has added to this theory is that this exhaustion is possibly present in the visceral and peripheral adipose compartments. The finding that adipocyte hypertrophy shows a strong association with IR, T2D, and MetS further suggests that at the cellular level, the hypothesized adipose tissue energy storage exhaustion could be due to limitations of the process of adipocyte proliferation, *i.e.*, adipogenesis. It is the adipogenesis limitation that histologically manifests as adipocyte hypertrophy.

Several previous studies have investigated adipocyte size and its association with various IR, T2D, and MetS-related phenotypic traits in South Asians. Anand et al. reported larger subcutaneous adipocyte size, higher fasting insulin, body fat content, ectopic liver fat, and lower adiponectin and HDL levels in South Asians as compared to Caucasians (36). However, when corrected for adipocyte size, these racial differences in fasting insulin and adiponectin were attenuated. Additionally, when corrected for fat distribution and adipocyte size, the racial difference in ectopic liver fat content was also attenuated. McLaren et al. reported a higher percentage of larger and lesser percentage of smaller adipocytes in subcutaneous adipose tissue in South Asians as compared to White Europeans (37). Weight gain in South Asians is associated with a greater impairment in insulin sensitivity and a smaller reduction in the number of small adipocytes. In other words, these findings indicate that their capability to store fat by increasing the size of adipocytes is limited. Liu et al. reported that omental adipocytes, though smaller than subcutaneous adipocytes, but their size shows a stronger correlation with measures of adiposity and metabolic parameters (38). The findings of the present study are consistent with their findings and additionally highlight the role of lower limb adipocyte hypertrophy also on the same lines.

Adipocyte hypertrophy and associated infiltration with macrophages with altered polarity have traditionally been considered to result from impaired adipogenesis. Previously, we conducted transcription profiling across different adipose tissue compartments in diabetics versus non-diabetics. Our findings revealed that several differentially expressed genes converge on pathways related to adipogenesis and inflammation, irrespective of the anatomical location of the tissue (19, 20). To gain deeper insights into the molecular mechanisms driving this adipose tissue pathology, we mapped the identified genes to their upstream and downstream regulatory networks, including transcription factors and protein kinases (39). This analysis highlighted the involvement of pathways associated with adipogenesis. Therefore, these studies provide a cellular and molecular framework for the findings of the present study, implicating adipocyte hypertrophy and inflammatory state in the pathogenesis of diabetes. In summary, all these findings suggest that heightened diabetes and MetS risk in South Asians is possibly due to impaired adipogenesis in their various adipose compartments, particularly the abdominal visceral and gluteo-femoral depots. Therefore, enhancing adipogenesis could be a useful therapeutic strategy in the management of T2D in this population.

There are several limitations of this study. The small sample size and different number of individuals in comparison groups’ cross-sectional design and the practical limitations in acquiring both thigh and abdominal adipose tissue samples from different subgroups of individuals are limiting factors in this study. Also, the measurement of insulin resistance with its surrogate HOMA-IR has its limitations. The participants of this study were undergoing an elective surgical procedure, so they may not be representative of the general population. Studies with large sample size, using a two-step hyperinsulinemic-euglycemic clamp combined with a glucose tracer for the estimation of insulin resistance and the use of newer technology of multi-omics instead of a microarray are needed to firmly establish the role of the FAT in the pathogenesis of diabetes specifically in thin fat Asian Indians.

## Conclusion

Among the adipose tissue-related traits examined, adipocyte hypertrophy in visceral and gluteo-femoral compartments, along with ectopic liver fat accumulation, showed the strongest associations with IR, T2D, and MetS. While quantitative traits such as visceral and lower limb fat mass were also relevant, these likely serve as proxies for deeper qualitative dysfunctions in adipose biology, particularly adipocyte expansion capacity.

Impaired adipogenesis—the failure to recruit and differentiate new, metabolically active adipocytes—emerges as a central defect underpinning this dysfunction. When subcutaneous depots lose their capacity to undergo hyperplasia in response to energy excess, existing adipocytes undergo pathological hypertrophy. This leads to mechanical stress, hypoxia, inflammation, and extracellular matrix remodeling—all hallmarks of adiposopathy.

Therapeutically, promoting healthy adipogenesis offers a promising strategy to offset ectopic fat accumulation and mitigate metabolic risk. Interventions that restore or preserve the formation of small, insulin-sensitive adipocytes—such as PPARγ agonists (e.g., low-dose thiazolidinediones), mesenchymal stem cell–based therapies, or agents targeting WNT and BMP signaling pathways—may enhance adipose tissue expandability and improve systemic glucose homeostasis. Additionally, lifestyle strategies such as calorie moderation and exercise are known to rejuvenate adipocyte turnover and improve depot-specific functionality, further reinforcing this therapeutic axis.

A key limitation of the present study was the restricted availability of MRI and adipocyte size data, which may have influenced the precision of regional fat analyses. Also, the overall cohort size was adequate (n = 322), certain subgroup analyses—such as visceral macrophage phenotyping (n = 22)—were limited by small sample sizes. This constraint may affect statistical robustness and generalizability of those specific findings, which should be interpreted with caution.

Future work integrating longitudinal imaging, functional adipogenesis assays, and omics-guided pathway interrogation may provide deeper insight into the therapeutic potential of adipose remodeling in South Asians.

## Data Availability

The raw data supporting the conclusions of this article will be made available by the authors, without undue reservation.
